# Intravenous administration exosomes derived from human amniotic mesenchymal stem cells improves neurological recovery after acute traumatic spinal cord injury in rats

**DOI:** 10.22038/ijbms.2024.76532.16576

**Published:** 2024

**Authors:** Honglong Zhou, Ji Wang, Peng Zhao, Dongsheng Le, Shanshan Cai, Guohua Mao

**Affiliations:** 1 Department of Neurosurgery, The Second Affiliated Hospital of Nanchang University, Nanchang 330006, China; 2 Department of Neurosurgery, Institute of Neuroscience, The Second Affiliated Hospital of Guangzhou Medical University, Guangzhou 510260, China; 3 Interventional Department, The Second Affiliated Hospital of Nanchang University, Nanchang 330006, China; 4 Department of Health Insurance, The First Affiliated Hospital of Nanchang University, Nanchang 330006, China; # These authors contributed eqully to this work

**Keywords:** Angiogenesis, Exosomes, Mesenchymal stem cells, Neural regeneration, Recovery of function

## Abstract

**Objective(s):**

Our previous study has showed that human amniotic mesenchymal stem cells (hAMSCs) transplantation improves neurological recovery after traumatic spinal cord injury (TSCI) in rats. However, less is known about the effects of exosomes derived from hAMSCs for TSCI. Here, we investigated whether hAMSCs-derived exosomes improve neurological recovery in TSCI rats and the underlying mechanisms.

**Materials and Methods:**

A rat traumatic spinal cord injury (TSCI) mode was established using a weight drop device. At 2 hr after TSCI, rats were administered either hAMSCs-derived exosomes or phosphate buffered saline via the tail vein. Locomotor recovery was evaluated by an open-field locomotor rating scale and gridwalk task. Spinal cord water content, hematoxylin and eosin (H&E) staining, Evans blue (EB) dye extravasation, immunofluorescence staining, and enzyme-linked immunosorbent were performed to elucidate the underlying mechanism.

**Results:**

hAMSCs-derived exosomes significantly reduced the numbers of ED1^+^ macrophages/microglia and caspase-3+cells and decreased the levels of reactive oxygen species, myeloperoxidase activity and inﬂammatory cytokines, such as tumor necrosis factor alpha, interleukin-6 and interleukin-1β. In addition, hAMSCs-derived exosomes significantly attenuated spinal cord water content and Evans blue extravasation, and enhanced angiogenesis and axonal regeneration. Finally, hAMSCs-derived exosomes also significantly reduced the lesion volume, inhibited astrogliosis, and improved functional recovery.

**Conclusion:**

Taken together, these findings demonstrate that hAMSCs-derived exosomes have favourable effects on rats after acute TSCI, and that they may serve as an alternative cell-free therapeutic approach for treating acute TSCI.

## Introduction

Traumatic spinal cord injury (TSCI) arises from concussive force or direct trauma to the spinal cord caused by spine fracture suffered in motor vehicle crashes, violence, sports injury, and falls, often resulting in paralysis and essential physiological dysfunction in the vast majority of patients (1). Moreover, the costs of treatment and healthcare rehabilitation are huge, which result in substantial social and economic burdens on affected individuals and their families (2). TSCI has become a major public health issue worldwide. TSCI induces complex and multifaceted pathophysiology processes (3). Current clinical therapies cannot achieve satisfactory results. Hence, it is critically important to develop new effective therapeutics for TSCI.

In recent years, with the rapid development of stem cell biology, stem cell-based therapies have been shown to be a promising therapeutic approach for TSCI. Stem cells from different tissue sources can provide therapeutic benefits in TSCI animal models by reducing inflammation and neuronal apoptosis, attenuating blood-spinal cord barrier disruption, immunomodulation, and enhancing angiogenesis and axon regeneration (4). Despite the encouraging results, there are still some limitations to the therapeutic application of stem cells such as immune rejection, tumorigenicity, low rates of transplanted cell survival and differentiation, poor cell retention rate, ethical problems, and so on (5-7). In addition, stem cells may be unsuitable for acute therapeutic intervention due to need for a massive time, specialized facilities, and equipment for cell culture and expansion. Furthermore, increasing studies have indicated that stem cells act in a paracrine manner rather than in a cellular manner (8). Exosomes secreted from stem cells play a significant role in paracrine effects, and they may constitute a replacement for cell-based therapy (9). Similar to the delivery of intact stem cells, equivalent or superior therapeutic effects were observed after the delivery of stem cell-derived exosomes (10, 11). 

Exosomes are nanovesicles, measuring 40 to 160 nm in diameter, which are secreted by various cells including stem cells. They play a vital role in mediating intercellular communication by transporting various molecules including proteins and microRNAs (miRNAs), among others (12). As a cell-free product, when compared with parental stem cells, exosomes provide several potential advantages, such as easy storage and transport, small size to facilitate the crossing of barriers, easy application via convenient and noninvasive routes, and no carcinogenic potential. In addition, recent studies have found that exosomes derived from mesenchymal stem cells (MSCs) promote motor function recovery in rats after spinal cord injury (13, 14). Therefore, exosomes derived from stem cells may be a promising cell-free clinical therapeutic alternative for TSCI (15). 

We have previously shown that either intraspinal or intravenous transplantation of human amniotic mesenchymal stem cells (hAMSCs) to TSCI rats results in improved functional recovery (16, 17). However, whether hAMSCs-derived exosomes promote functional recovery in rats with TSCI and the potential underlying mechanism remain elusive.

Hence, the goal of this study is to determine whether intravenous administration of hAMSCs-derived exosomes promotes functional recovery in rats with TSCI and investigate the underlying mechanism.

## Materials and Methods

***Isolation, culture, and differentiation of hAMSCs***

Human amniotic membranes from cesarean sections of women were collected after written informed consent. The mean gestational age of collection was 38 ± 1.5 weeks. The procedure was approved by the Ethics Committee of The Second Affiliated Hospital of Guangzhou Medical University. hAMSCs were harvested from amniotic membranes by enzymatic digestion method according to our previously described method (16). The cells were incubated at 37 °C in a humidified atmosphere with 5% CO_2_. When cells reached 80% conﬂuence, they were split at a ratio of 1:3. Von Kossa, Alcian Blue, and Oil Red O staining were used to detect the differentiation ability of hAMSCs after osteogenic, chondrogenic, and adipogenic induction, respectively.

***Isolation, characterization, and labeling of exosomes***

Passage 4 hAMSCs were cultured and grown to 60%–80% confluence. After removing the culture medium, cells were rinsed three times with PBS and cultured in exosome-depleted FBS for an additional 24 hr. Exosomes from the collected supernatants were purified by differential ultracentrifugation as previously described. The isolation procedures were performed at 4 °C. The culture supernatant was first centrifuged at 500g for 10 min to remove cells and cellular debris. Exosomes were collected by ultracentrifugation of the supernatant at 100,000g for 70 min, and then the final pellet was resuspended in sterile PBS and ultracentrifuged at 100,000g for another 70 min at 4 °C. Exosome protein concentration was measured using a BCA Protein Assay Kit (Thermo Fisher Scientific, USA). The expression of CD9, CD63, and CD81 was measured using western blotting. The following primary antibodies were used: CD81, CD63, and CD9 (1:1000 for all; Abcam, USA). The morphology of exosomes was examined with transmission electron microscopy (TEM). A nanoparticle tracking analysis system was used to measure the size distribution and concentration of exosomes. 

***Animal and experimental groups***

A total of one hundred and four female adult Sprague-Dawley rats (230–260 g) were acquired from the Vital River Company in Beijing, China. The animals were kept in a 12-hr light/dark cycle with *ad libitum* access to food and water. The experiments were conducted in an environment with a steady temperature of 23±3 °C and humidity of 52%±10%. Experiments involving animals were approved by the Animal Experimental Ethical Committee of The Second Affiliated Hospital of Guangzhou Medical University. Rats were randomly allocated into two groups: the hAMSCs-derived exosomes group (n=52) and the control group (n=52).

***Establishment of TSCI model and transplantation of hAMSCs-derived exosomes***

Animals were anesthetized by intraperitoneal injection of pentobarbital sodium (Sigma-Aldrich, 100 mg/kg). The spinal cord was exposed by a laminectomy performed at the T8-T10 level. TSCI was produced by dropping a 10-g metal weight from a height of 2.5 cm using an NYU impactor. Following the surgical procedure, the wound was immediately sutured. At 2 hr after injury, 200 μg exosomes (roughly 1×10^6^ hAMSCs) in 200 µl PBS were injected via the tail vein. Equal volumes of PBS were administrated as a control. Bladders of rats with TSCI were manually expressed two times every day until autonomous bladder function was restored.

***Immunofluorescence and histological analyses***

At 3,7, and 28 days after TSCI, rats were deeply anesthetized with sodium pentobarbital by intraperitoneal administration and then transcardially perfused with 0.9% saline followed by 4% paraformaldehyde. Subsequently, the spinal cords were promptly extracted and immersed in 4% paraformaldehyde for 24 hr. Then tissues were dehydrated with 30% sucrose for 72 hr. The tissues were embedded in OCT. Frozen sections of 20-µm thickness were cut using a cryostat. Immunofluorescence staining was carried out by incubating the sections overnight at 4 °C with specific primary antibodies: GAP-43 (1:500; Millipore, USA), ED1 (1:100; Millipore, USA), caspase-3 (1:50; Abcam, UK), human nuclear protein (hNu; 1:100; Millipore, USA), serotonin (5-HT; 1:2000; Sigma, USA), Willebrand factor (vWF; 1:1000; Abcam, USA). The secondary antibody used species-specific fluorophore-conjugated immunoglobulin. Some slides were mounted using an antifade reagent containing DAPI (Sigma). Fluorescent images were obtained using a confocal microscope. At 28 days after TSCI, some slides were stained with HE. 

***Measurements of spinal cord water content***

The spinal cord water content was measured at 7 days after TSCI using the wet-dry weight method. After rats were deeply anesthetized with sodium pentobarbital, the spinal cords were removed immediately, and 15-mm-sized spinal tissues were obtained at the edge of the injury site. Spinal tissues placed on a pre-weighed piece of aluminum foil were measured on an electronic analytical balance to give the wet weight and then dried in an electric oven for 48 hr at 95 °C to obtain the dry weight. The spinal cord water content was calculated as follows:(wet weight-dry weight)/(wet weight) × 100%.

***Evans blue (EB) method for quantitative analysis of BSCB disruption***

At 7 days after TSCI, the BSCB permeability was quantified by EB extravasation. In brief, 2% EB was administered via the femoral vein. After 2 hr, rats were sacrificed with an overdose of sodium pentobarbital by intraperitoneal administration and perfused with saline. The spinal cords were extracted and immediately weighed, then immersed in methanamide at 60 °C for 24 hr. After centrifugation, supernatant was collected to determine EB concentration. Spectrophotometry was utilized to detect the EB content at 620 nm. The result was presented as µg/g of spinal cord tissue.

***Measurement of myeloperoxidase (MPO) activity in spinal cord tissues***

MPO is an enzyme from the azurophilic granula of neutrophil granulocytes. MPO is used as a marker of the activation state or presence of neutrophils. MPO activity was measured using an MPO assay kit (Nanjing Jiancheng Bio-engineering Institute, China) according to the manufacturer’s protocol at 7 days after TSCI. The result was expressed as units/g protein.

***ELISA***

At 7 days after TSCI, rats were deeply anesthetized, and then the spinal segments (1 cm long) around the lesion center were obtained. The spinal cord tissue was cut into small pieces and homogenized. Homogenates were then centrifuged at 4 °C at 1000 rpm. The concentrations of TNF-α, IL-1β, and IL-6 were detected using ELISA kits (R&D Systems) in accordance with the manufacturer’s protocol.

***Measurement of reactive oxygen species (ROS) levels***

At 7 days after TSCI, the ROS levels in the spinal cord samples were determined using a ROS Assay Kit (Jiancheng, Nanjing, China) according to the manufacturer’s protocols. After the rats were deeply anesthetized, a 0.5-cm segment was obtained from the spinal cord injury site. The tissue samples were homogenized in cold PBS and then centrifuged at 1000 g for 10 min at 4 °C. Supernatants were incubated with DCFH-DA in a 96-well plate for 30 min at 37 °C in the dark. Total protein concentrations were measured using a BCA Protein Assay Kit (Thermo Fisher Scientific, USA). Final measurements of fluorescent intensity were read with a microplate reader at Ex/Em=490/520 nm and presented as fluorescence/mg protein.

***Lesion volume analysis***

HE staining was used to distinguish the lesion and spared tissue, in HE stained sections at 28 days after TSCI, the lesion area was then outlined and quantified by ImageJ software. The lesion volume for each rat was calculated by the sum of the total lesion area multiplied by the intersection distance (about 200 μm) according to a previously published method.

***BBB open ﬁeld locomotor test and Gridwalk test***

At 1, 7, 14, 21, and 28 days after TSCI, locomotor function was tested using the BBB scale. The BBB score ranges from 0 (complete hindlimb paralysis) to 21 (normal locomotion). The BBB test was carried out by two independent evaluators who were unaware of group assignments. 

A grid walk test was performed to evaluate deficits in descending motor control after TSCI. Briefly, the rats were allowed to cross a 1-m-long runway with irregularly assigned gaps (0.5–5 cm) between round metal bars, where the number of footfall errors was defined as the inability to grasp a bar and instead fell between them, which was counted in each crossing, and a mean error rate was calculated. Every rat crossed the grid at least three times. The gridwalk test was carried out at 1, 7, 14, 21, and 28 days after TSCI by two independent evaluators. 

***Statistical analyses***

Statistical analyses were performed with SPSS 18.0. The results were presented as mean ± SEM. Statistical analysis for behavioral test data was conducted using repeated measures ANOVA. For other data, the analysis was performed by independent samples *t* test if they were in normal distribution. Otherwise, the analysis was performed with the Mann-Whitney U test. A value of *P*<0.05 was considered statistically significant.

## Results

***Characters of hAMSCs and hAMSCs-derived exosomes***

**Figure 1:**
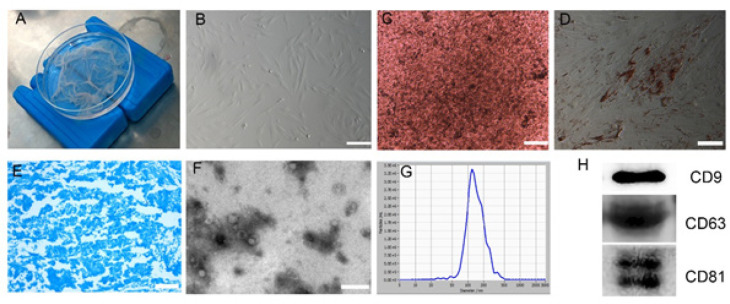
Morphology and identification of hAMSCs and hAMSCs-derived exosomes

Human amniotic membranes showed a thin translucent membrane (Figure 1A). Cultured hAMSCs were found to have fibroblast-like morphology under light microscopy after four population doublings (Figure 1B). hAMSCs were next cultured in various types of differentiation medium to identify their differentiation potential. After culture in the osteogenic differentiation medium, hAMSCs were chemically induced to differentiate into the osteogenic lineage, as identified by von Kossa staining (Figure 1C). After culture in the adipogenic differentiation medium, hAMSCs were induced to differentiate into the adipogenic lineage, as identified by Oil Red O staining (Figure 1D). After culture in the chondrogenic differentiation medium, hAMSCs were induced to differentiate into the chondrogenic lineage, as identified by alcian blue staining (Figure 1E). Overall, the results showed that hAMSCs have the potential to differentiate into all three germ layers. 

hAMSCs-derived exosomes were next isolated and purified from culture supernatants by ultracentrifugation, and the morphology and markers of hAMSCs-derived exosomes were identified using TEM and WB. Results showed that isolated hAMSCs-derived exosomes revealed saucer-shaped vesicle morphology, and a diameter almost ranging from between 50 and 120 nm (Figure 1G). WB analysis indicated that hAMSCs-derived exosomes expressed several exosome markers CD9, CD63, and CD81 (Figure 1H).

Photographic image of isolated human amniotic membrane (A). Representative images showing the spindle-like morphology of hAMSCs(B). hAMSCs can differentiate into osteoblasts, adipocytes, and chondrocytes by von Kossa(C), Oil Red O(D), and Alcian Blue staining(E) after osteogenic, adipogenic, and chondrogenic induction *in vitro*, respectively. Representative image showing the saucer-shaped vesicle morphology of hAMSCs-derived exosomes observed with TEM(F). The concentration and size distribution of hAMSCs-derived exosomes by NTA(G). WB analysis of CD9, CD63, and CD81 expression in hAMSCs-derived exosomes(H). Scale bar=100 µm (B-E)and 500 nm(F).

***Tracking of hAMSCs-derived exosomes in vivo***

**Figure 2:**
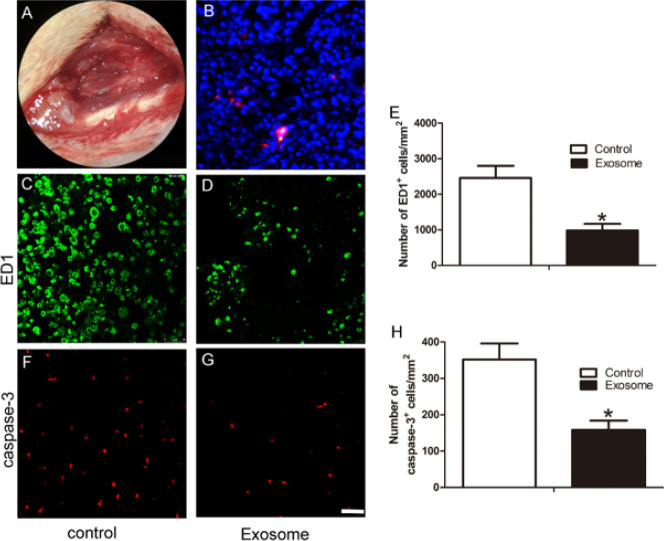
hAMSCs-derived exosomes inhibited the inflammatory apoptosis after TSCI

To track transplanted hAMSCs-derived exosomes *in vivo*, we labeled hAMSCs-derived exosomes with the fluorescent cell-tracking dye CM-DiI *in vitro* before transplantation in TSCI rats (N=3). The results showed that DiI-labelled exosomes were detected in the injured spinal cord at 3 days after TSCI (Figure 2B).

Representative figure showing crushed site after the surgical procedures under operating microscope (A). Representative figure showing DiI-labelled hAMSCs-derived exosomes were found in the injured spinal cord at 3 days after TSCI (B). Representative immunofluorescence staining images (C, D) and quantitative analysis of ED1+ cells at the epicenter of the injury at 7 days after TSCI(E). Representative immunofluorescence staining images (F, G) and quantitative analysis of caspase-3+ cells at the epicenter of the injury at 7 days after TSCI (H). n=4 per group. All the data are presented as mean±SEM. *P<0.05 versus the control group. Scale bar=50 µm.

***hAMSCs-derived exosome administration reduced in*** ***ﬂ*** ***ammation and apoptosis***

We constructed a TSCI rat model (Figure 2A) and treated it with hAMSCs-derived exosomes. It was tested whether treatment with hAMSCs-derived exosomes affects TSCI-induced inﬂammation and apoptosis. At 7 days after TSCI, compared with the control group, the number of ED1+ cells of the hAMSCs-derived exosomes group was significantly lower (*P*<0.05) (Figure 2C–E), and the number of caspase-3^+^ apoptotic cells of the hAMSCs-exosomes group was significantly lower (*P*<0.05) (Figure 2F–H). 

***hAMSCs-derived exosome administration reduced*** ***the levels of inflammatory factors, MPO activity, and*** ***ROS levels***

**Figure 3:**
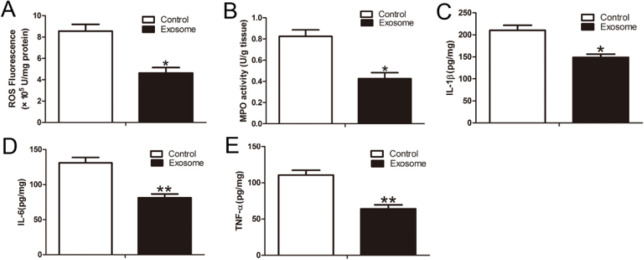
hAMSCs-derived exosomes reduced the levels of ROS, MPO activity, and proinflammatory cytokines

At 7 days after TSCI, ROS levels were significantly lower in the hAMSCs-derived exosomes group than in the control group (*P*<0.05) (Figure 3A). Similarly, MPO activity was significantly lower in the hAMSCs-derived exosomes group than in the control group (*P*<0.05) (Figure 3B). We next measured the levels of the major inflammatory cytokines IL-6, TNF-a, and IL-1β in the injured spinal cord. The results showed that the levels of proinﬂammatory IL-1β, IL-6, and TNF-a were significantly lower in the hAMSCs-derived exosomes group than the control group (*P*<0.05 and *P*<0.01, respectively; Figure 3C–E). 

ROS was measured by the ROS Assay Kit at 7 days after TSCI. MPO activity was measured using an MPO assay kit at 7 days after TSCI. Analysis of the levels of ROS (A). Analysis of the levels of MPO activity(B). ELISA analysis of IL-1β (C), IL-6(D), and TNF-α (E) levels in injury spinal cord tissues at 7 days after TSCI. n=6 per group. All the data are expressed as mean±SEM. *P<0.05, **P<0.01 versus the control group.

***hAMSCs-derived exosome administration reduced spinal cord water content and BSCB leakage***

**Figure 4:**
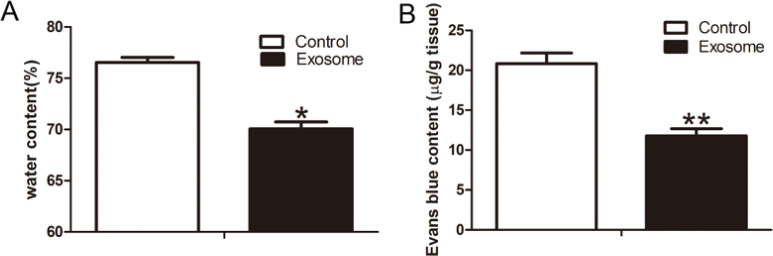
hAMSCs-derived exosomes reduced BSCB leakage and spinal cord water content at 7 days after TSCI

At 7 days after TSCI, the spinal cord water content was measured using the wet-dry weight method, with results showing that the spinal cord water content was significantly reduced in the hAMSCs-derived exosomes group compared to the control group (*P*<0.05) (Figure 4A). BSCB permeability was assessed by EB. The EB content in the spinal cord tissue of rats in the hAMSCs-derived exosomes group was significantly reduced compared to the control group (*P*<0.01) (Figure 4B). These results showed that intravenous administration of hAMSCs-derived exosomes attenuated BSCB leakage and reduced spinal cord water content after TSCI.

***hAMSCs-derived exosome administration promoted angiogenesis and axonal regeneration and inhibited astrogliosis***

**Figure 5:**
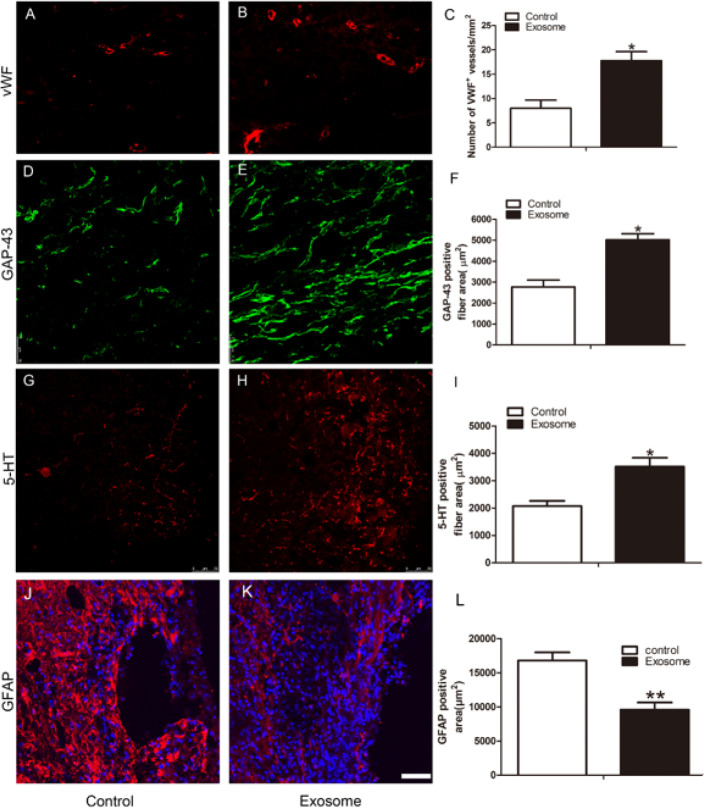
hAMSCs-derived exosomes enhanced angiogenesis and axonal regeneration at 28 days after TSCI

To determine the degree of angiogenesis, vWF^+^ vessels at the injury site were measured at 28 days after TSCI. The number of vWF+ vessels was significantly higher in the hAMSCs-derived exosomes group than in the control group (*P*<0.05) (Figure 5A-C). Axonal regeneration is important for spinal cord repair and neurological recovery. GAP-43, a neuronal growth cone marker, is associated with axonal sprouting and outgrowth in re-growing axons (18). Immunofluorescence results demonstrated a significant increase in the area of GAP-43+ fibers in the hAMSCs-derived exosomes group compared to the control group (*P*<0.05) (Figure 5D-F). 5-HT fibers, which originate in the brainstem nuclei and descend to the spinal cord, play a prominent role in hindlimb movements (19). 5-HT+ neuronal fiber areas in the spinal cord tissue of the hAMSCs-derived exosomes group were obviously higher than in the control group (*P*<0.05) (Figure 5G-I). Astrogliosis in the TSCI lesion site at 28 d after TSCI was evaluated by immunostaining for GFAP (marker of astrocytes) and quantified the density of GFAP expression in the lesion boundary region. GFAP^+^ area at the boundary of the injury of the hAMSCs-derived exosomes group showed a significant decrease compared to the control group (*P*<0.01, Figure 5J–L). Those results demonstrated that hAMSCs-derived exosome administration promoted angiogenesis and axonal regeneration, and inhibited astrogliosis.

***hAMSCs-derived exosome administration reduced the lesion volume and improved locomotion recovery***

**Figure 6:**
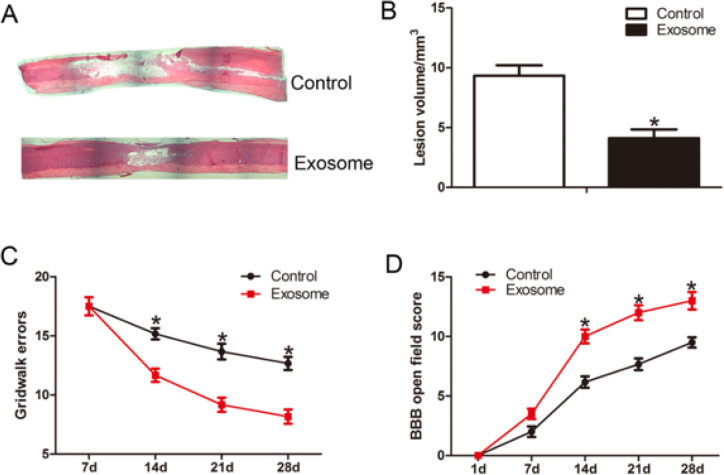
hAMSCs-derived exosomes reduced the lesion volume and improved neurological outcome after TSCI

We tested whether hAMSCs-derived exosome administration could reduce the lesion volume. At 28 days after TSCI, the sections were stained with HE, and the lesion volume was examined under a light microscope. The results indicated that hAMSCs-derived exosome administration significantly decreased the lesion volume compared with the control group (*P*<0.05) (Figure 6A-B).

We evaluated the effect of hAMSCs-derived exosome administration on functional recovery after TSCI by using the BBB scale and Gridwalk test. The results showed that the BBB scores of the hAMSCs-derived exosomes group were significantly higher than those of the control group from 14 days to the time point thereafter after SCI (*P*<0.05) (Figure 6D). Consistent with BBB score analysis, from 14 days to 28 days after TSCI, the hAMSCs-derived exosomes group had a significantly lower gridwalk footfall error rate than the control group (*P*<0.05) (Figure 6C).

hAMSCs: human amniotic mesenchymal stem cells

hAMSCs: human amniotic mesenchymal stem cells; TSCI: traumatic spinal cord injury

hAMSCs: human amniotic mesenchymal stem cells; ROS: reactive oxygen species; MPO: myeloperoxidase

hAMSCs: human amniotic mesenchymal stem cells; TSCI: traumatic spinal cord injury; BSCB: blood-spinal cord barrier

Representative immunofluorescence images and quantitative analysis of vWF+ blood vessels (A-C). Representative immunofluorescence images and quantitative analysis of GAP-43+ fiber areas (D-F). Representative immunofluorescence staining images and quantitative analysis of 5-HT+ fiber areas (G-I). Representative immunofluorescence staining images and quantitative analysis of GFAP+ areas (J-L). n=4 per group. All the data are presented as mean±SEM. *P<0.05, **P<0.01 versus the control group. Scale bar=50 µm.

hAMSCs: human amniotic mesenchymal stem cells; TSCI: traumatic spinal cord injury

Representative images of HE staining to measure lesion volume (A). Quantitative analysis of lesion volume (B). Motor function was assessed by the Gridwalk scores(C) and BBB scores (D). n=6 per group at each time point. All values are expressed as mean±SEM. *P<0.05 versus the control group.

hAMSCs: human amniotic mesenchymal stem cells; TSCI: traumatic spinal cord injury; HE: hematoxylin-eosin; BBB: basso, Beattie and Bresnahan

## Discussion

In the present study, we investigated the therapeutic effects of intravenous administration of hAMSCs-derived exosomes in a TSCI rat model. Our study showed that intravenous administration of hAMSCs-derived exosomes after TSCI significantly decreased inflammatory cell infiltration and apoptosis, and suppressed excessive astrogliosis. hAMSCs-derived exosomes significantly reduced ROS and inﬂammatory cytokines levels, including TNF-a, IL-6, and IL-1β, reduced spinal cord water content and BSCB leakage, and enhanced angiogenesis and axonal regeneration. Additionally, hAMSCs-derived exosomes significantly reduced lesion volume and eventually improved locomotor recovery.

 In our previous study, the results showed no indication that the grafted hAMSCs differentiated into glial or neural cells in the injured spinal cord (16). These findings were consistent with other studies indicating that paracrine mechanisms rather than cellular replacement must be responsible for the therapeutic potential of MSCs (20). In recent years, numerous studies have shown that MSCs-derived exosomes may be a key paracrine factor for the repair of injured tissues (21). In the present study, we successfully isolated, purified, and identified hAMSCs-derived exosomes. Intravenous administration of hAMSCs-derived exosomes resulted in the exosomes reaching the injured spinal cord, indicating that hAMSCs-derived exosomes could easily cross the BSCB and subsequently provide therapeutic value.

Neuroinflammation is a vital component of secondary responses after spinal cord injury. During the secondary injury phase after spinal cord injury, multiple lines of evidence suggest substantial inflammatory cells such as macrophages and neutrophils migrate to the lesion site. These cells then secrete inflammatory cytokines including TNF-a, IL-6, and IL-1β, and generate reactive oxygen species, resulting in increased spinal cord damage and reduced axonal regeneration (22, 23). Therefore, alleviating inflammatory reaction contributes to the functional recovery of the damaged spinal cord (24, 25). Interestingly, previous studies showed that stem cell-derived exosomes inhibited the inflammatory reaction and improved locomotor recovery (26, 27). Similarly, our results demonstrated that hAMSCs-derived exosomes significantly decreased MPO activity, inﬂammatory cytokine levels, and the number of ED1^+^ activated macrophages/microglia compared with the controls. These results indicated that the therapeutic effects of hAMSCs-derived exosomes may be partly related to a mechanism associated with reducing neuroinflammation.

Astrogliosis is a biological process whereby resident astrocytes in the central nervous system change into a reactive state after CNS injury, which plays a vital in the pathology of spinal cord injury (28, 29). Although astrogliosis has a number of neuroprotective functions associated with restricting inflammation and repairing the primary damage following spinal cord injury, excessive astrogliosis causes secretion of inflammatory cytokines and axon growth inhibitory factors to cause inflammation and prevent axon regeneration (30, 31). Thus, suppressing excessive astrogliosis may represent a potential strategy for treating spinal cord injury. Numerous studies have shown that stem cell transplantation can inhibit astrogliosis, thereby improving functional recovery after SCI (32-34). In our study, results indicated that the GFAP+ expression rate was significantly decreased in the hAMSCs-derived exosomes group compared with the control group. These results suggested that hAMSCs-derived exosomes suppressed excessive astrogliosis and were in part responsible for neurological recovery.

ROS plays a vital in mediating physiological and pathophysiological functions of the central nervous system (35). However, excessive levels of ROS after spinal cord injury, mainly produced by damaged mitochondria, NADPH oxidases, inflammatory cells, and the Fenton reaction, can cause damage to proteins, lipids, and DNA, resulting in axonal degeneration, demyelination, and neuronal cell apoptosis (36, 37). Many studies have revealed that ROS scavengers can attenuate neuronal cell injury, and improve locomotor recovery following spinal cord injury (38, 39). Therefore, the scavenging of ROS is thought to be a potential essential strategy for TSCI treatment. In this study, we observed that the ROS levels of the hAMSCs-derived exosomes group were significantly reduced compared with the control group. In addition, the number of caspase-3^+^ apoptotic cells in the hAMSCs-derived exosomes group was significantly lower compared with the control group. These results are not surprising, because previous studies showed that hAMSCs-derived exosomes have the ability to reduce ROS generation (40).

After TSCI, vascular disruption occurs immediately and triggers a cascade of pathological processes that result in tissue damage and neurological deficits (41). Angiogenesis, the growth of new blood vessels from preexisting microvasculature, establishes a new microenvironment that provides nutrients and oxygen to support tissue repair and regeneration after injury (42). In addition, study reported that promoting angiogenesis in the area of SCI may contribute to neuronal remodeling and axonal regeneration (43). In our study, the number of vWF+ blood vessels was significantly higher in the hAMSCs-derived exosomes group than in the control group. Moreover, the area of GAP-43^+^ fibers at the epicenter of the injury in the hAMSCs-derived exosomes group was significantly higher than in the control group. 5-HT^+^ fibers areas in the caudal lumbar region of the hAMSCs-derived exosomes group were significantly higher than in the control group. These data are consistent with other studies that demonstrate MSCs-derived exosomes promote angiogenesis and axonal regeneration (44, 45).

Similar to the blood-brain barrier, the BSCB is composed of vascular endothelial cells, pericytes with tight/adherens junction protein complexes, astrocytes, and a basement membrane, which forms a physical and biochemical barrier preventing neurotoxic products from systemic circulation to the central nervous system (46, 47). TSCI can result in BSCB disruption, which allows blood cells, detrimental factors, macrophages, and other inflammatory cells to enter the area of injury, causing local edema, ischemia, focal hemorrhage, and inflammation, finally leading to permanent neurological deficits (48). Therefore, early repair of the BSCB is considered essential for limiting damage after TSCI. In our previous study, intravenous administration of hAMSCs significantly reduced BSCB disruption, as assessed by Evans blue method (17). In the present study, our results indicated that intravenously administered hAMSCs-derived exosomes also significantly reduced the extravasation of EB dye compared with controls. These results show that hAMSCs-derived exosomes reduced BSCB disruption.

This study has some limitations: First, the intrinsic off-target effects caused by non-specific uptake of exosomes in other tissues may lead to potential adverse effects or complications. Second, this study was carried out in small animals, larger animal models of TSCI such as non-human primates may be useful tools in facilitating the development of translational therapies for human TSCI.

## Conclusion

Taken together, it can be concluded that hAMSCs-derived exosomes promoted the functional recovery of TSCI rats by reducing neuroinflammation, cell apoptosis, astrogliosis, and lesion volume, protecting the BSCB, and enhancing angiogenesis and axonal regeneration. These results suggest that hAMSCs-derived exosomes can provide multi-therapeutic effects against multi-destructive pathways caused by TSCI. Thus, intravenous administration of hAMSCs-derived exosomes appears to be an effective therapeutic approach for acute TSCI.
